# Supplementary material for: Gender policy and intimate partner violence in Colombia
Source: PLoS One. 2023 Nov 1;18(11):e0290313. doi: 10.1371/journal.pone.0290313 (PMC10619832; doi:10.1371/journal.pone.0290313)
Supplement: S1 File — (DOCX) [file pone.0290313.s001.docx]

**S1: Treatment, and choice of treated and untreated departments**

My hypothesis is that the new and amended laws and national gender policies, implemented roughly between 2008 and 2013, combined with the departments' existing, or newly adopted gender policies, affected IPV in 2015. The new laws were, according to Hoyos and Benjuméa [1], a turning point in the fight against IPV. And a large part of a long list of actions included in the national development plan, [2], and the national gender policy plan, were implemented [3].

Although it is possible that previous efforts to reduce IPV had an effect, there seems to be a general agreement that this was negligible [2-5]. There are several reasons why policies failed to have an impact, but a major reason was the policymakers' and civil servants' lack of interest [3]. In any case, if there was an impact already in 2010, this would bias the results downwards.

One assumption is thus that the policies implemented in 2008-2010, such as the new law and the adoption of gender policy programs, had little or no impact on IPV at the time of collection of the baseline data, which took place between November 2009 and November 2010. The basis for this assumption is that there is a considerable time lag between the formal decision to adopt a gender policy program and the appearance of any visible effects on IPV; it is likely to be a question of at least three years or more. After a formal decision is taken, there is a need to set up an institutional and implementation framework, including a department for women's affairs or a gender office (secretaría de la mujer). Then there is the question of design, financing, and implementation of activities, which are based on a multi-sectoral approach cutting across both state and non-state actors at various levels. In the department Atlántico, for example, it took a year from the adoption of the gender policy program before the gender office was in place [6]. One year later, in 2013, the gender office had not managed to coordinate policies with other local government offices [7]. To this should it be added that a gender policy program consists of many activities, out of which those aimed at IPV constitute a minor part. Thus, prompt implementation of the relevant policies might take time even when the institutions are in place. To appreciate the comprehensiveness of the programs, see, for example, the programs of the Government of Chocó [8], or the Republic of Kenya [9], which is similar and in English. In the revised version of the paper, I show that the results get weaker when I add departments that adopted gender programs after 2011.

Given this, four issues related to identification should be considered. First, two departments had adopted gender programs well before 2010, Antioquia and Bogota D.C. However, Bogota’s program was mainly a list of intentions with no financial support and opposition from civil servants[10]. A new program was therefore launched in 2010 [8], which I consider a proper program.

The fact that Antioquia had a program in place raises the issue of staggered treatment: as Goodman-Bacon [11] and others show, staggered treatment can cause biases if ignored. However, my hypothesis is that it was the combined effect of new national policies and gender policy at the department level that affected IPV. Thus, the fact that Antioquia had a gender policy program in place before this period is immaterial, its treatment occurs in 2010. In any case, the statistical problem of staggered treatment is likely to be minor when it is a question of only one unit.

Second, I base the choice of treated departments on AECID [12], a review of regional gender policies in Colombia carried out by the Spanish Agency for International Development Cooperation. It provides a ranking of the departments up until late 2011, based on the presence of gender policy programs and institutional settings related to gender policies. Eight departments (including Bogota D.C.) had gender policy programs in place; two were in the process of adopting programs, and four departments had gender offices but no program. AECID also reports the on the quota law, requiring that at least 30% of the members of the cabinet should be females. In the main analysis, I consider departments with a gender policy program, or a gender office, as treated. All these departments fulfilled the quota requirement. There is one potential borderline case, Caquetá, which fulfilled the quota requirement, planned to adopt a gender policy program, and had one person working with gender issues, but no proper gender office. It adopted a gender policy program in 2017. However, as described below, Caquetá is excluded from the main analysis because of other concerns.

Third, a few departments approved gender policy programs after the completion of AECID but before 2015. Since I only have information from one survey after treatment, i.e., 2015, I cannot address issues related to their staggered rollout for these departments. Nevertheless, given the brief period between the adoption of the programs and the 2015 survey, it is unlikely that they had a strong effect on IPV. As mentioned, in the robustness section I report estimates when these departments are considered treated.

Fourth, the adoption of gender policy has not been random. Unfortunately, I have not been able to find systematic information or studies on why the departments differ in this respect. Nevertheless, apart from political will, resources, and capacity are likely to be crucial factors, since developing a comprehensive gender policy framework is a major effort, including many meetings and the involvement of stakeholders. The process usually takes several years [7, 10]. For example, Caquetá where in the process of implementing a gender policy in 2011 according to AECID (12), but adopted it in 2017 [8].

# **References**

1. Hoyos CA, Benjumea A. Las medidas de protección a mujeres víctimas de violencia: Análisis de la ley 1257 de 2008 y recomendaciones para su efectividad. Corporación Humanas Centro Regional de Derechos Humanos y Justicia de Género. 2016:1-16.

2. Government of Colombia. Equidad de Género para las Mujeres: Documento CONPES Social 161 Bogota: Consejo Nacional de Política Económica y Social 2013.

3. Proyectamos Colombia SAS. Evaluación institucional y de resultados con enfoque participativo de la Política de Equidad de Género para las Mujeres, de acuerdo con lo previsto en el CONPES 161 de 2013. Bogotá: Proyectamo Colombia SAS; 2017.

4. Gómez López C, Murad R, Calderón MC. Historias de violencia, roles, prácticas y discursos legitimadores. Violencia contra las mujeres en Colombia 2000-2010. Bogotá: Ministerio de Salud; 2013.

5. Ministerio de Salud. No más cifras de violencia contra la mujer Bogotá2017 [Available from: <https://www.minsalud.gov.co/Paginas/No-mas-cifras-de-violencia-contra-la-mujer.aspx>.

6. Government of Atlántico. Nace la Secretaría de las Mujeres en el Atlantico 2014 [Available from: <https://www.atlantico.gov.co/index.php/noticias/prensa-mujer/1997-nace-la-secretaria-de-las-mujeres-en-el-atlantico>.

7. Díaz LS, De los Ríos Castiblanco JC. Evaluación de la política pública de la mujer en el departamento del atlántico en el año 2013. Saber, Ciencia y Libertad. 2016;11(2):55-72.

8. Government of Chocó. Politica Pública de Equidad de Género para Mujeres Chocoanas. Gobernación del Chocó; 2017.

9. Government of Kenya. National Policy on Gender and Development Towards Creating a Just, Fair and Transformed Society Free from Gender Based Discrimination and All Spheres of Life Practices. Nairobi; 2019.

10. Barón Mesa GF, Muñoz Eraso JP. Aciertos y fallas en la implementación de la Política de Mujeres y Equidad de Género de Bogotá. Papel Político. 2016;21(1):101-20.

11. Goodman-Bacon A. Difference-in-differences with variation in treatment timing. Journal of Econometrics. 2021;225(2):254-77.

12. AECID. Ciudades e Igualdad de Género. Agencia Española de Cooperación Internacional para el Desarrollo; 2011.
