# Supplementary material for: Gender policy and intimate partner violence in Colombia
Source: PLoS One. 2023 Nov 1;18(11):e0290313. doi: 10.1371/journal.pone.0290313 (PMC10619832; doi:10.1371/journal.pone.0290313)
Supplement: S2 File — (DOCX) [file pone.0290313.s002.docx]

**S2: Differences between treated and untreated departments**

It is noteworthy that no department in eastern and southern Colombia had a gender policy program or a proper gender office in place in 2011: in one department, Caquetá, one person worked with gender issues. Thus, all treated departments are in the northern and western parts of the country (the Andes and along the Caribbean and Pacific coast). Moreover, no department in eastern and southern Colombia is included in DHS 2000, while all the others are included in all DHSs.

The departments in eastern and southern Colombia differ by having small populations compared to the others, as Figure A1 shows. Since they have large areas, the number of inhabitants per square kilometre is also tiny (not reported). The size of the population probably matters in the adoption of gender policies: one reason is that a department with a large population is likely to have a larger government, and a higher capacity to implement new policies.

Another reason possible reason is differences in resources. Thus, income, or GDP, may also play a crucial role since it should be correlated with government income. Figure A2 shows that GDP per capita is somewhat higher on average in treated departments, but that there is a great deal of variation in both groups. Consequently, GDP per capita is unlikely to be a key determinant of the adoption of gender policies.

Figure A1. Population in 2010. Small departments in eastern and southern Colombia are in black stripes, untreated departments in blue, and treated departments in black.

Source: National Administrative Department of Statistics of Colombia [1].

Figure A2. GDP per capita, 2010. Small departments in eastern and southern Colombia and not included in DHS 2000 in black stripes, untreated departments in blue, and treated departments in black

Source: National Administrative Department of Statistics of Colombia [2].

Yet another factor is the efficiency or quality of the provincial government. The Economic Commission for Latin America and the Caribbean publishes an indicator of financial management and public management efficiency [3]. The index runs from 0 to 100. Figure A3 shows that there are clear differences across the departments but that the average score for the treated ones only is somewhat higher than for the others if the departments from eastern and southern Colombia are excluded.

Colombia has experienced armed conflict for a long time, which is likely to have affected IPV directly [4, 5], and possibly the political will and strength to fight it. Figure A4 reports an index of the incidence of armed conflict, measured over the period 2002-2013. The index is composed of six indicators (armed action, kidnappings, forced migration, cultivation of coca, violent murders, and victims of landmines) and runs from 0 to 6. There is not a clear difference between treated and untreated departments, particularly if we exclude the eastern and southern ones. After 2013, the index drops to zero in all departments, because of the peace process, initiated in 2012 [6].

Economic inequality might also be associated with both IPV and the adoption of gender policies. Figure A5 reports Gini coefficients for 24 departments. Economic inequality is high in all departments, over 0.45, and the averages for the treated and untreated groups are similar, 0.53 and 0.52. Thus, economic inequality is unlikely to be of major concern for the study. Moreover, it changes slowly and should not affect the trajectories of IPV prevalence.

Substantial changes in GDP can result in unemployment and economic stress, which could affect IPV. Thus, Figure A6 depicts the percentage change in real GDP between 2010 and 2015. Most departments experienced substantial growth, 15-35%. On average, treated departments had a higher growth rate than untreated departments, but this is due to the decrease in economic activity in Chocó; without Chocó the untreated grew by a couple of percentage points more than the treated.

Figure A3. Indicator of public management efficiency in 2009. Departments in eastern and southern Colombia are in black stripes, untreated departments in blue color, and treated departments in black color.

Source: CEPAL [3].

Figure A4. Index of armed conflict (0-5) for the period 2002-2013. Departments in eastern and southern Colombia not included in DHS 2000 in black stripes, untreated departments in blue color, and treated departments in black color.

Source: Government of Colombia [6].

Figure A5. Gini coefficients for 2010. Departments in eastern and southern Colombia are in black stripes, untreated departments in blue color, and treated departments in black.

Source: National Administrative Department of Statistics of Colombia [7]. No data are available for seven departments in eastern and southern Colombia.

Figure A6. Department GDP growth 2010-2015. Small departments in eastern and southern Colombia and not included in DHS 2000 in black stripes, untreated departments in blue color, and treated departments in black.

Source: National Administrative Department of Statistics of Colombia [2].

To conclude, there are substantial differences between treated departments and some of the untreated ones. Most noteworthy, all the departments in eastern and southern Colombia lack gender policy programs and proper gender offices. Although their GDP per capita is not systematically lower than in other departments, their small populations imply limited resources and few public employees, which might have constrained policymakers in implementing gender policies. It is also possible that there is less public pressure for change since there are few individuals in a large geographical area, as well as other, unobservable, differences between them and the other departments. In the main analysis, I, therefore, exclude the eight departments located in eastern and southern Colombia, though the evaluation of parallel trends and the results are only affected marginally by keeping them.

**References**

1. DANE. Demographic statistics: Departamento Administrativo Nacional de Estadística, Government of Colombia; 2022 [Available from: <https://www.dane.gov.co/index.php/estadisticas-por-tema/demografia-y-poblacion>.

2. DANE. National Accounts: Departamento Administrativo Nacional de Estadística, Government of Colombia; 2022 [Available from: <https://www.dane.gov.co/index.php/estadisticas-por-tema/cuentas-nacionales/cuentas-nacionales-departamentales>.

3. Ramírez Jaramillo JC, Parra-Peña RI. Escalafón de la competitividad de los departamentos en Colombia, 2009. Bogotá: United Nations, CEPAL; 2010. Report No.: 9211218152.

4. Rieckmann J. Violent conflicts increase the risk of domestic violence in Colombia. DIW Economic Bulletin. 2014;4(12):23-6.

5. Svallfors S. Hidden casualties: The links between armed conflict and intimate partner violence in Colombia. Stockholm Research Reports in Demography; 2020.

6. Government of Colombia. Índice de incidencia del conflicto armado. Bogota D.C.: Departamento Nacional de Planeación – Grupo de Proyectos Especiales; 2016.

7. DANE. Inequality: Departamento Administrativo Nacional de Estadística, Government of Colombia; 2022 [Available from: <https://www.dane.gov.co/index.php/estadisticas-por-tema/pobreza-y-condiciones-de-vida/pobreza-y-desigualdad>.
