# Supplementary material for: Gender policy and intimate partner violence in Colombia
Source: PLoS One. 2023 Nov 1;18(11):e0290313. doi: 10.1371/journal.pone.0290313 (PMC10619832; doi:10.1371/journal.pone.0290313)
Supplement: S3 File — (DOCX) [file pone.0290313.s003.docx]

**S3: Complete Table 3, Differences-in-Differences regressions**

Table A1. Effects of gender policy on intimate partner violence during the past year, all women currently and formerly in a union. Physical and/or sexual violence

|  | (1) | (2) | (3) | (4) |
| --- | --- | --- | --- | --- |
| VARIABLES | IPV past year  23 departments | IPV past year  23 departments | IPV past year  31 departments | IPV past year  31 departments |
|  |  |  |  |  |
| Post x Policy | -0.048*** | -0.050*** | -0.049*** | -0.047*** |
|  | (0.011) | (0.010) | (0.010) | (0.010) |
| Post | 0.013 | -0.028 | 0.012* | -0.019 |
|  | (0.008) | (0.017) | (0.007) | (0.015) |
| Age (base 15-19) |  |  |  |  |
| 20-24 |  | 0.021 |  | 0.021 |
|  |  | (0.014) |  | (0.013) |
| 25-29 |  | -0.025* |  | -0.023* |
|  |  | (0.013) |  | (0.012) |
| 30-34 |  | -0.032** |  | -0.031** |
|  |  | (0.013) |  | (0.012) |
| 35-39 |  | -0.055*** |  | -0.054*** |
|  |  | (0.013) |  | (0.012) |
| 40-44 |  | -0.072*** |  | -0.071*** |
|  |  | (0.013) |  | (0.012) |
| 45-49 |  | -0.083*** |  | -0.083*** |
|  |  | (0.013) |  | (0.013) |
| Education (base none) |  |  |  |  |
| Incomplete primary |  | -0.010 |  | -0.010 |
|  |  | (0.015) |  | (0.014) |
| Complete primary |  | -0.014 |  | -0.016 |
|  |  | (0.015) |  | (0.014) |
| Incomplete secondary |  | 0.001 |  | -0.001 |
|  |  | (0.015) |  | (0.014) |
| Complete secondary |  | -0.046*** |  | -0.047*** |
|  |  | (0.015) |  | (0.014) |
| Higher |  | -0.057*** |  | -0.058*** |
|  |  | (0.015) |  | (0.015) |
| Father beat mother |  | 0.079*** |  | 0.079*** |
|  |  | (0.005) |  | (0.005) |
| Wealth index (base Poorest) |  |  |  |  |
| Poorer |  | 0.013 |  | 0.012 |
|  |  | (0.009) |  | (0.008) |
| Middle |  | -0.009 |  | -0.010 |
|  |  | (0.011) |  | (0.010) |
| Richer |  | -0.040*** |  | -0.041*** |
|  |  | (0.012) |  | (0.012) |
| Richest |  | -0.069*** |  | -0.070*** |
|  |  | (0.013) |  | (0.012) |
| Ethnic group (base none below) |  |  |  |  |
| Afro-Colombian |  | 0.022*** |  | 0.022*** |
|  |  | (0.008) |  | (0.008) |
| Indigenous |  | 0.009 |  | 0.007 |
|  |  | (0.011) |  | (0.010) |
| Palaquero/Rom/Raizal |  | -0.015 |  | -0.014 |
|  |  | (0.054) |  | (0.053) |
| Household size |  | 0.002 |  | 0.002 |
|  |  | (0.001) |  | (0.001) |
| Urban |  | 0.065*** |  | 0.065*** |
|  |  | (0.009) |  | (0.008) |
| Index armed conflict |  | -0.029*** |  | -0.020*** |
|  |  | (0.006) |  | (0.005) |
| Log GDP/capita |  | -0.021 |  | -0.008 |
|  |  | (0.058) |  | (0.048) |
| 8 department | -0.028** | -0.059*** | -0.028** | -0.049*** |
|  | (0.013) | (0.020) | (0.013) | (0.017) |
| 11 department | 0.023** | 0.019 | 0.023** | 0.018 |
|  | (0.012) | (0.029) | (0.012) | (0.025) |
| 13 department | 0.033** | 0.011 | 0.033** | 0.014 |
|  | (0.015) | (0.016) | (0.015) | (0.016) |
| 15 department | 0.020 | -0.011 | 0.020 | -0.005 |
|  | (0.015) | (0.016) | (0.015) | (0.015) |
| 17 department | -0.024 | -0.038 | -0.024 | -0.032 |
|  | (0.016) | (0.024) | (0.016) | (0.021) |
| 19 department | 0.013 | -0.008 | 0.013 | -0.004 |
|  | (0.016) | (0.040) | (0.016) | (0.034) |
| 20 department | -0.032** | -0.052*** | -0.032** | -0.048*** |
|  | (0.014) | (0.016) | (0.014) | (0.015) |
| 23 department | -0.011 | -0.055 | -0.011 | -0.041 |
|  | (0.012) | (0.040) | (0.012) | (0.034) |
| 25 department | 0.025* | -0.007 | 0.025* | -0.001 |
|  | (0.014) | (0.014) | (0.014) | (0.014) |
| 27 department | 0.074*** | 0.024 | 0.074*** | 0.031 |
|  | (0.017) | (0.051) | (0.017) | (0.044) |
| 41 department | 0.024 | 0.004 | 0.024 | 0.008 |
|  | (0.018) | (0.024) | (0.018) | (0.022) |
| 44 department | -0.043*** | -0.073** | -0.043*** | -0.063** |
|  | (0.015) | (0.034) | (0.015) | (0.029) |
| 47 department | -0.045*** | -0.082** | -0.045*** | -0.073** |
|  | (0.011) | (0.041) | (0.011) | (0.035) |
| 50 department | 0.047*** | 0.072 | 0.047*** | 0.053 |
|  | (0.017) | (0.046) | (0.017) | (0.039) |
| 52 department | 0.008 | 0.001 | 0.008 | 0.003 |
|  | (0.014) | (0.054) | (0.014) | (0.047) |
| 54 department | 0.024* | 0.001 | 0.024* | 0.003 |
|  | (0.014) | (0.034) | (0.014) | (0.030) |
| 63 department | -0.006 | -0.038 | -0.006 | -0.025 |
|  | (0.014) | (0.029) | (0.014) | (0.025) |
| 66 department | -0.011 | -0.025 | -0.011 | -0.020 |
|  | (0.013) | (0.022) | (0.013) | (0.019) |
| 68 department | -0.001 | -0.007 | -0.001 | -0.009 |
|  | (0.014) | (0.035) | (0.014) | (0.030) |
| 70 department | -0.045*** | -0.099** | -0.046*** | -0.081** |
|  | (0.013) | (0.048) | (0.013) | (0.040) |
| 73 department | 0.017 | -0.001 | 0.017 | 0.003 |
|  | (0.014) | (0.023) | (0.014) | (0.020) |
| 76 department | 0.031*** | 0.021* | 0.031*** | 0.021* |
|  | (0.011) | (0.011) | (0.011) | (0.011) |
| 18 department |  |  | 0.059*** | 0.049 |
|  |  |  | (0.015) | (0.045) |
| 81 department |  |  | 0.019 | 0.016 |
|  |  |  | (0.020) | (0.026) |
| 85 department |  |  | -0.002 | -0.018 |
|  |  |  | (0.017) | (0.044) |
| 86 department |  |  | 0.001 | 0.000 |
|  |  |  | (0.017) | (0.031) |
| 94 department |  |  | -0.022 | -0.063 |
|  |  |  | (0.022) | (0.049) |
| 95 department |  |  | 0.038** | 0.009 |
|  |  |  | (0.017) | (0.052) |
| 97 department |  |  | 0.100*** | 0.047 |
|  |  |  | (0.027) | (0.065) |
| 99 department |  |  | 0.020 | -0.022 |
|  |  |  | (0.019) | (0.045) |
|  |  |  |  |  |
| Observations | 46,156 | 44,518 | 56,461 | 54,350 |
| R-squared | 0.005 | 0.034 | 0.005 | 0.033 |

Note: Weights for domestic violence are used. Robust standard errors clustered at the department level in parentheses, *** p<0.01, ** p<0.05, * p<0.1.

Table A2. Effects of gender policy on intimate partner violence during the past year, all women currently and formerly in a union. Physical and/or sexual violence

|  | (1) | (2) | (3) | (4) |
| --- | --- | --- | --- | --- |
|  |  |  |  |  |
| VARIABLES | IPV ever  23 departments | IPV ever  23 departments | IPV ever  31 departments | IPV ever  31 departments |
|  |  |  |  |  |
| Post x Policy | -0.055*** | -0.058*** | -0.054*** | -0.055*** |
|  | (0.013) | (0.012) | (0.012) | (0.011) |
| Post | -0.002 | 0.042** | -0.003 | 0.043** |
|  | (0.009) | (0.021) | (0.009) | (0.018) |
| Age (base 15-19) |  |  |  |  |
| 20-24 |  | 0.064*** |  | 0.064*** |
|  |  | (0.015) |  | (0.014) |
| 25-29 |  | 0.057*** |  | 0.058*** |
|  |  | (0.014) |  | (0.014) |
| 30-34 |  | 0.076*** |  | 0.078*** |
|  |  | (0.015) |  | (0.014) |
| 35-39 |  | 0.084*** |  | 0.084*** |
|  |  | (0.015) |  | (0.014) |
| 40-44 |  | 0.071*** |  | 0.072*** |
|  |  | (0.015) |  | (0.015) |
| 45-49 |  | 0.093*** |  | 0.093*** |
|  |  | (0.015) |  | (0.015) |
| Education (base none) |  |  |  |  |
| Incomplete primary |  | 0.007 |  | 0.009 |
|  |  | (0.019) |  | (0.018) |
| Complete primary |  | 0.007 |  | 0.008 |
|  |  | (0.018) |  | (0.018) |
| Incomplete secondary |  | 0.024 |  | 0.024 |
|  |  | (0.018) |  | (0.018) |
| Complete secondary |  | -0.048*** |  | -0.047*** |
|  |  | (0.019) |  | (0.018) |
| Higher |  | -0.092*** |  | -0.089*** |
|  |  | (0.019) |  | (0.019) |
| Father beat mother |  | 0.141*** |  | 0.140*** |
|  |  | (0.006) |  | (0.006) |
| Wealth index (base Poorest) |  |  |  |  |
| Poorer |  | 0.023** |  | 0.021** |
|  |  | (0.010) |  | (0.009) |
| Middle |  | -0.004 |  | -0.005 |
|  |  | (0.012) |  | (0.012) |
| Richer |  | -0.038*** |  | -0.040*** |
|  |  | (0.013) |  | (0.013) |
| Richest |  | -0.083*** |  | -0.084*** |
|  |  | (0.015) |  | (0.015) |
| Ethnic group (base none below) |  |  |  |  |
| Afro-Colombian |  | 0.038*** |  | 0.038*** |
|  |  | (0.010) |  | (0.010) |
| Indigenous |  | 0.015 |  | 0.013 |
|  |  | (0.013) |  | (0.012) |
| Palaquero/Rom/Raizal |  | -0.095 |  | -0.092 |
|  |  | (0.065) |  | (0.064) |
| Household size |  | 0.005*** |  | 0.005*** |
|  |  | (0.001) |  | (0.001) |
| Urban |  | 0.096*** |  | 0.095*** |
|  |  | (0.010) |  | (0.010) |
| Index armed conflict |  | 0.003 |  | 0.005 |
|  |  | (0.007) |  | (0.006) |
| Log GDP/capita |  | -0.123* |  | -0.124** |
|  |  | (0.072) |  | (0.058) |
| 8 department | -0.069*** | -0.104*** | -0.069*** | -0.102*** |
|  | (0.015) | (0.024) | (0.015) | (0.021) |
| 11 department | 0.020 | 0.077** | 0.020 | 0.080*** |
|  | (0.014) | (0.035) | (0.014) | (0.030) |
| 13 department | -0.041** | -0.065*** | -0.040** | -0.063*** |
|  | (0.017) | (0.018) | (0.017) | (0.017) |
| 15 department | 0.045** | 0.033* | 0.045** | 0.035* |
|  | (0.019) | (0.019) | (0.019) | (0.019) |
| 17 department | -0.058*** | -0.102*** | -0.058*** | -0.101*** |
|  | (0.020) | (0.029) | (0.020) | (0.026) |
| 19 department | 0.034* | -0.058 | 0.035* | -0.060 |
|  | (0.019) | (0.047) | (0.019) | (0.040) |
| 20 department | -0.080*** | -0.105*** | -0.080*** | -0.104*** |
|  | (0.017) | (0.019) | (0.017) | (0.018) |
| 23 department | -0.091*** | -0.168*** | -0.091*** | -0.168*** |
|  | (0.016) | (0.050) | (0.016) | (0.042) |
| 25 department | 0.052*** | 0.031 | 0.052*** | 0.033* |
|  | (0.020) | (0.019) | (0.020) | (0.019) |
| 27 department | 0.072*** | -0.072 | 0.073*** | -0.074 |
|  | (0.018) | (0.063) | (0.018) | (0.053) |
| 41 department | 0.030 | -0.017 | 0.030 | -0.018 |
|  | (0.020) | (0.028) | (0.020) | (0.025) |
| 44 department | -0.116*** | -0.180*** | -0.115*** | -0.180*** |
|  | (0.018) | (0.041) | (0.018) | (0.034) |
| 47 department | -0.081*** | -0.177*** | -0.081*** | -0.178*** |
|  | (0.016) | (0.051) | (0.016) | (0.043) |
| 50 department | 0.082*** | 0.140** | 0.082*** | 0.138*** |
|  | (0.021) | (0.058) | (0.021) | (0.048) |
| 52 department | 0.043** | -0.084 | 0.043** | -0.089 |
|  | (0.018) | (0.066) | (0.018) | (0.056) |
| 54 department | -0.022 | -0.116*** | -0.022 | -0.118*** |
|  | (0.017) | (0.042) | (0.017) | (0.036) |
| 63 department | -0.054*** | -0.111*** | -0.054*** | -0.108*** |
|  | (0.016) | (0.036) | (0.016) | (0.030) |
| 66 department | -0.050*** | -0.093*** | -0.049*** | -0.092*** |
|  | (0.016) | (0.027) | (0.016) | (0.023) |
| 68 department | -0.049*** | 0.023 | -0.049*** | 0.025 |
|  | (0.018) | (0.042) | (0.018) | (0.036) |
| 70 department | -0.140*** | -0.243*** | -0.140*** | -0.242*** |
|  | (0.017) | (0.060) | (0.017) | (0.048) |
| 73 department | 0.028* | -0.021 | 0.028* | -0.021 |
|  | (0.017) | (0.028) | (0.017) | (0.025) |
| 76 department | 0.028** | 0.019 | 0.028** | 0.019 |
|  | (0.014) | (0.013) | (0.014) | (0.013) |
| 18 department |  |  | 0.022 | -0.110** |
|  |  |  | (0.016) | (0.054) |
| 81 department |  |  | 0.000 | -0.005 |
|  |  |  | (0.019) | (0.026) |
| 85 department |  |  | -0.035* | 0.040 |
|  |  |  | (0.019) | (0.053) |
| 86 department |  |  | 0.005 | -0.092** |
|  |  |  | (0.020) | (0.037) |
| 94 department |  |  | -0.102*** | -0.221*** |
|  |  |  | (0.025) | (0.059) |
| 95 department |  |  | 0.027 | -0.163*** |
|  |  |  | (0.021) | (0.063) |
| 97 department |  |  | 0.131*** | -0.061 |
|  |  |  | (0.033) | (0.079) |
| 99 department |  |  | -0.029 | -0.158*** |
|  |  |  | (0.018) | (0.053) |
| Observations | 46,156 | 44,518 | 56,461 | 54,350 |
| R-squared | 0.012 | 0.053 | 0.011 | 0.053 |

Note: Weights for domestic violence are used. Robust standard errors clustered at the department level in parentheses, *** p<0.01, ** p<0.05, * p<0.1.
