# Supplementary material for: Gender policy and intimate partner violence in Colombia
Source: PLoS One. 2023 Nov 1;18(11):e0290313. doi: 10.1371/journal.pone.0290313 (PMC10619832; doi:10.1371/journal.pone.0290313)

**S4: Unfaithfulness and IPV ever**

Figure A7. Predictive margins for IPV ever and unfaithfulness based on the triple differences model.


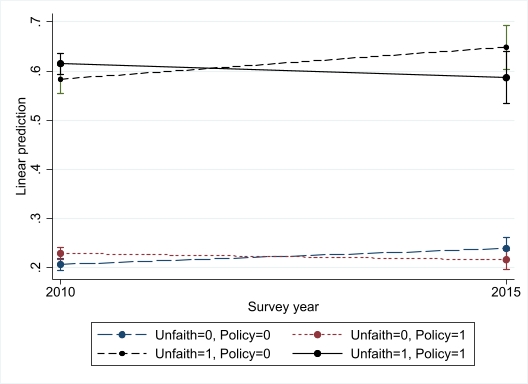

Supplement: S4 File — (DOCX) [file pone.0290313.s004.docx]
