# Supplementary material for: Gender policy and intimate partner violence in Colombia
Source: PLoS One. 2023 Nov 1;18(11):e0290313. doi: 10.1371/journal.pone.0290313 (PMC10619832; doi:10.1371/journal.pone.0290313)
Supplement: S5 File — (DOCX) [file pone.0290313.s005.docx]

**S5: Assumption of parallel trends**

To evaluate the assumption of parallel trends, I use data from the DHS 2000 and DHS 2005, in addition to DHS 2010 and DHS 2015. There is a DHS from 1995, but it does not have the same questions about exposure to IPV as the others. The DHS from 2000 also differs somewhat from the more recent ones by requesting different responses, i.e., the alternatives are “Algunas veces” and “Nunca” (never), while they are “Alguna vez“, “Nunca”, and “El ultimo año” (last year) in the more recent surveys. The response “Algunas veces” implies more than once while “Alguna vez” means (at least) once. In addition, the DHS 2000 does not have information about IPV during the past year, it only covers 23 out of the 32 departments, and it has much fewer observations for the departments surveyed i.e., 7 000 compared to over 20 000.

Figure A8 plots the group-level trajectories (left panel) and the results obtained with a linear-trends model (right panel), which imply a common reference point for the first survey year, for the combination of IPV ever and 23 departments. The figure shows almost identical trends between 2005 and 2010**.**

Figures A9 and A10 report event study coefficients and their 95% confidence intervals for models of “IPV ever” and “IPV last 12 months” for the 23 departments over the periods 2000-2015 and 2005-2015, respectively. The coefficient for 2010 is set to zero. All three pretreatment coefficients are close to zero and insignificant.

Figure A8. Graphical diagnostics for parallel trends 2005-2015, IPV ever, 31 departments .

**
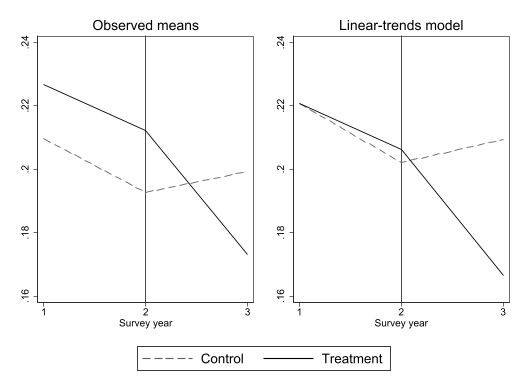
**

Figure A9. Event study, 2000-2015, IPV ever, 23 departments, estimated coefficients, and 95% confidence intervals.

**
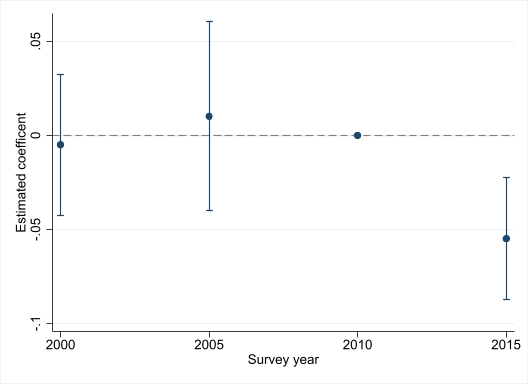
**

Figure A10. Event study, 2005-2015, IPV past year 23 departments, estimated coefficients, and 95% confidence intervals.

**
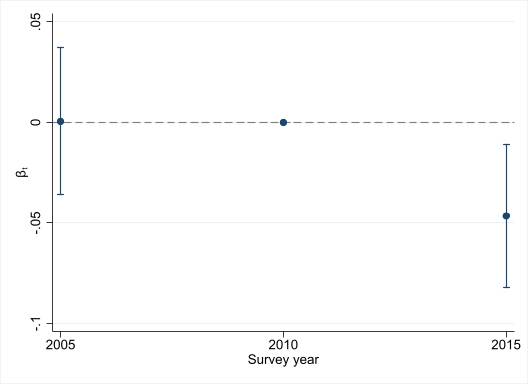
**

Table A3 reports F-tests for the pretreatment period for the three models. The null hypothesis of parallel trends is not rejected in any of the three tests.

Table A3. Tests of linear parallel trend

| IPV past 12 months 2005-2015, 23 departments | | IPV past 12 months 2005-2015, 31 departments | | IPV ever 2000-2015, 23 departments | |
| --- | --- | --- | --- | --- | --- |
| F(1, 22) = 0.07  Prob > F = 0.791 |  | F(1, 30) = 0.11  Prob > F = 0.742 |  | F(1, 22) = 0.02  Prob > F = 0.890 |  |

Note: Based on event study models with control variables.

I use the procedures developed by Rambachan and Roth [1] to show how two types of violations of the parallel trend assumption affect the significance of the estimates. Figure A11 reports robust 95% confidence intervals of the DiD estimates for IPV ever when deviations of the parallel trends are assumed to be a multiple of the maximal deviation observed in the pre-treatment period. When Mbar is equal to zero, i.e., Mbar=original, the confidence interval is obtained under the assumption of exactly parallel trends, when Mbar=1, the deviation from parallel trends is assumed to be the same as the maximal deviation in the pre-treatment period, when Mbar=2, the deviation is assumed to be twice as large as in the pre-treatment period, and so on. The estimate holds for violations of the parallel trends assumption in the post-treatment period that are twice as large as those observed during the pre-treatment period, the breakdown value for a significant effect is Mbar==2.5.

The other violation, denoted M, can be viewed as an evaluation of shocks in the post-treatment period that generate changes in the linear trend. M is the percentage points change per period in the difference between the trends of the two groups. This creates a non-linear deviation from the parallel trend. In Figure A12, which reports the results for IPV ever, M = 0 corresponds to the linear violation of parallel trends observed in the pre-treatment period, which is the same as Mbar=1 in Figure A11. The breakdown value for a significant effect is M=0.03, i.e., three percentage points per period.

Figure A11. Sensitivity analysis of IPV ever, violation of parallel trends in the post-treatment period of Mbar times maximum deviation in pretreatment period.


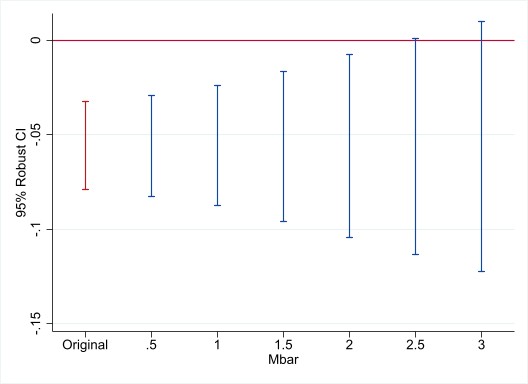


Note: The sample includes DHS 2005, DHS 2010, and DHS 2015. The following control variables are used: five-year age dummies, education, father beat mother, a wealth index, number of household members, urban residence, GDP per capita, index of armed conflict, and department fixed effects.

Figure A12. Sensitivity analysis of IPV ever, violation of parallel trends in the post-treatment period due to change in slope of M each period.


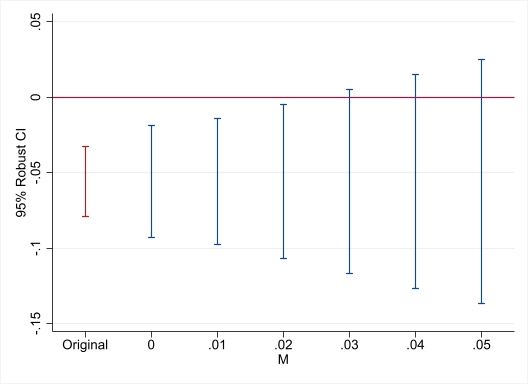


Note: See Figure A11.

# **References**

1. Rambachan A, Roth J. A More Credible Approach to Parallel Trends. Review of Economic Studies 2023; Forthcoming.
