# Supplementary material for: Gender policy and intimate partner violence in Colombia
Source: PLoS One. 2023 Nov 1;18(11):e0290313. doi: 10.1371/journal.pone.0290313 (PMC10619832; doi:10.1371/journal.pone.0290313)
Supplement: Attachment — Submitted filename: Responses to Reviewers.docx [file pone.0290313.s006.docx]

**Response to Reviewers**

**Reviewer 1**

The introduction actually looks like an expanded summary of the study. The most important thing is that it does not have an explicit objective.
Restructure the manuscript to the traditional structure, include an objective, and resubmit.

I apologize for the confusion. The structure of papers varies across disciplines, and since PLOS ONE is a multidisciplinary journal, I wrote the Introduction in a way that is common in my discipline. However, the paper is about global health, so I agree that the Introduction, in particular, should be changed. Moreover, the Introduction was not in line with the guidelines provided by PLOS ONE. I have now rewritten the Introduction and added a Discussion that contains the old Conclusion and some new material. I think the structure of the rest of the paper is clear, it is based on the content of the paper, which is more extensive than what is usually the case in papers in natural and health sciences.

**Reviewer 2**

1 Introduction: Remove the details pertaining to methods and findings.

I have improved the Introduction and removed the topics suggested.

2 Methods: Why fixed effects? Apply Hausman test to validate the decision. How the econometric method used handles the endogeneity- add explanation.

The difference-in differences (DiD) approach requires a treated group and a control group. In the simplest DiD specification, these are measured by a variable that is 1 for the treated units and 0 for the others. This variable is then interacted with a variable that is 1 during the treatment period and 0 otherwise to obtain the DiD variable. I use a more flexible specification where I allow the provinces to have separate intercepts; these are the fixed effects (I mention this because the terminology varies across disciplines). The fixed effects capture all omitted variables that change little or not at all during the study period and eliminate all data pooling. The models I estimate are called two-way fixed effects models because I also have time (survey) fixed effects. These models are frequently used when applying the DiD approach. Nevertheless, the results differ only slightly between the two specifications when one has few time periods, as in my case.

It is, of course, possible to use random effects instead of fixed effects, but then one risks getting biased estimates due to omitted variables. Moreover, random effects models partially pool the data, while the DiD approach focuses on changes within each unit over time (department in my case), and this is obtained by using fixed effects.

The issue of endogeneity is primarily related to the assumption of parallel trends, that is, that the outcomes of the treated and untreated groups would have followed the same trend on average if the treated group had not been treated. Of course, this is not testable; however, I evaluate the assumption as carefully as possible and check the robustness of the results.

The use of control variables primarily aims to remove possible (minor) differences between trends and convince the reader that the results are robust. Moreover, even when the parallel trends assumption holds without control variables, adding them can increase the precision of the estimates.

3. Empirical Model: You should also include variables like 'religion', 'gender inequality', and 'depression' to improve the specification.

This comment relates to the choice of control variables. Unfortunately, I do not have any information on religion or depression. I assume that most Columbians belong to the Catholic fate and that they did not see it as a variable of interest; a DHS has many questions. Moreover, education, wealth, urban/rural, and ethnic group variables are likely to capture the differences across religious groups, if any.

There is a depression module in the Columbian DHSs, but in contrast to many other DHSs, it is only used for the elderly.

I have been careful not to include variables that can be outcomes of the treatment, since they might lead to biased results. The programs evaluated aimed to reduce gender inequality, so gender inequality could have been a dependent variable. Indeed, a reduction in IPV is a reduction in gender inequality.

4 Table 1: Why 'no eduction' is repeated?

This was a mistake that I have corrected. It should have been higher education.

5 Information on the econometric method used is missing (I am not talking about DiD).

I assume you are referring to the estimator. I use OLS or, in other words, estimate linear probability models (LPMs). I now make this clear page 14 where I write

“The linear probability model, i.e., OLS, is used to estimate the DiD regressions. The DHS sample design is accounted for by using clustering and weights, as recommended (48).”

Although one could estimate logistical models, the nonlinearity makes it challenging to interpret the effects of the treatment, so they are normally not used. In any case, logistical and LPMs usually yield similar results.

6 Diagnostic statistics of the models are missing.

I agree that I do not perform any standard regression diagnostic tests. However, there is no point in testing for linearity, normality, and homoscedasticity, since the dependent variable is binary. Linearity is ”imposed,” as it were, and the residual is either 0 or 1. Since I use survey data, independence is dealt with using methods that adjust for the stratified samples, that is, by using clustering and weights, as recommended by the DHS. See page 14 and the reference to

Croft TN, Marshall AM, Allen CK, Arnold F, Assaf S, Balian S. Guide to DHS statistics. Rockville: ICF. 2018;645.

I evaluate leverage in Table 6 and the other key DiD assumptions by estimating event study models (Figs A9-10), simulating the effects of deviations from parallel trends (Figs 6-7), and alternating the definitions of the treatment and control groups (Tables 5 and 7).

For a brief description of the DID models, see https://www.publichealth.columbia.edu/research/population-health-methods/difference-difference-estimation
