# Supplementary material for: Gender policy and intimate partner violence in Colombia
Source: PLoS One. 2023 Nov 1;18(11):e0290313. doi: 10.1371/journal.pone.0290313 (PMC10619832; doi:10.1371/journal.pone.0290313)
Supplement: Attachment — Submitted filename: Response to Reviewers.docx [file pone.0290313.s007.docx]

Comment:

“The manuscript was not summarized and restructured as recommended in the first review round.
It contains 7 tables and 7 figures. It is not a thesis, it should have no more than 4 tables/figures. It was recommended to fit the manuscript to the traditional structure: Introduction, Methods, Results, Discussion, but it was not done. The author should have the ability to summarize the information he wants to show.”

I apologize for not understanding the width of the comments on the previous version of the paper; however, I did make substantial changes to the Introduction and Discussion/Conclusion.

I have created a section called Materials and methods with two subsections: Data and outcomes and Method. In addition, I have moved the data descriptive section and two other sections to the Results section, which now contains four sub-sections: Data description and summary statistics, Regression analysis, Indirect evidence, and Assumptions and robustness checks. I hope that this is in line with the comment above.

The paper is about twice as long as articles published in medical journals such as the Lancet, but compared to many papers in social sciences and economics, it is short (30 pages with doubly spaced text). In fact, one reason why I submitted the paper to Plosone is that it does not restrict the number of pages, tables, or figures. Moreover, I included several of the tables and figures in response to an earlier Plosone referee report, and I do not think the paper will be strengthened by moving them to the online appendix.
